# All-Possible-Couplings Approach to Measuring Probabilistic Context

**DOI:** 10.1371/journal.pone.0061712

**Published:** 2013-05-06

**Authors:** Ehtibar N. Dzhafarov, Janne V. Kujala

**Affiliations:** 1 Department of Psychological Sciences, Purdue University, West Lafayette, Indiana, United States of America; 2 Department of Mathematical Information Technology, University of Jyväskylä, Jyväskylä, Finland; University of Nottingham, United Kingdom

## Abstract

From behavioral sciences to biology to quantum mechanics, one encounters situations where (i) a system outputs several random variables in response to several inputs, (ii) for each of these responses only some of the inputs may “directly” influence them, but (iii) other inputs provide a “context” for this response by influencing its probabilistic relations to other responses. These contextual influences are very different, say, in classical kinetic theory and in the entanglement paradigm of quantum mechanics, which are traditionally interpreted as representing different forms of physical determinism. One can mathematically construct systems with other types of contextuality, whether or not empirically realizable: those that form special cases of the classical type, those that fall between the classical and quantum ones, and those that violate the quantum type. We show how one can quantify and classify all logically possible contextual influences by studying various sets of probabilistic couplings, i.e., sets of joint distributions imposed on random outputs recorded at different (mutually incompatible) values of inputs.

## Introduction

Consider a system with two inputs, 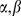, and two random outputs, 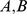, about which it is assumed that 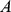 is *not* influenced by 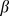, nor 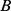 by 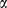. A necessary condition for this selectivity of influences is *marginal selectivity* [1]: changes in the values of 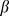 do not influence the distribution of 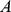, and analogously for 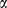 and 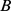. Let, for example, both inputs and outputs be binary: 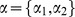, 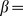 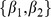, and 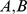 attain values 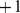 and 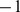 each. Denoting by 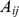 and 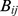 the two outputs conditioned on 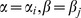 (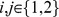), the distribution of 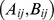 is described by the joint probabilities 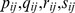 (summing to 1) in the matrix
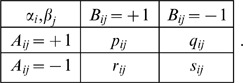
(1)Assuming all four combinations 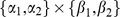 are possible, marginal selectivity in this example means
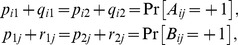
(2)for all 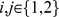.

The assumption of *selective influences*, however, is stronger. It requires that the joint distribution of the two outputs satisfies, for all 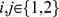,
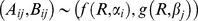
(3)where 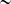 stands for “has the same distribution as,” 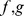 are some functions, and 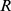 is a source of randomness that does not depend on 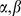 [2]–[8]. In our example (1) this means
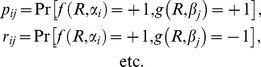
(4)In the quantum mechanical context (see below) 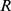 is interpreted as “hidden variables.” Such a representation may or may not exist when marginal selectivity is satisfied. For instance, the latter is satisfied in the following four distributions,
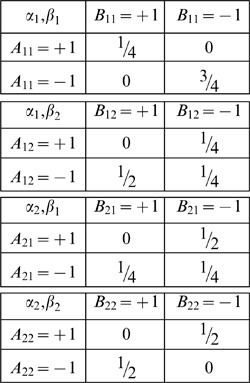
(5)It can be shown, however, that no representation (3) here is possible as the joint probabilities violate the Bell/CHSH inequalities considered below (Section 1 of Theory and Text S1). At the same time, a representation in the form of (3) is possible for the similar distributions
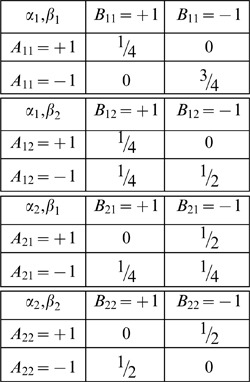
(6)One can think of 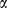 and 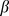 in (5) and (6) as being involved in different kinds of *probabilistic context* for the “direct” dependence of, respectively, 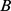 on 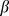 and 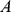 on 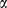.

We propose a principled way of quantifying and classifying conceivable contextual influences, whether within or outside the scope of (3). Our approach is neutral with respect to such issues as causality or what distinguishes direct influences from contextual. We merely accept as a given a diagram of direct input-output correspondences (e.g., 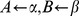) and study the joint distribution of the outputs at all possible values of the inputs. The interpretation of the diagram is irrelevant insofar as it is compatible with the observed pattern of marginal selectivity: as 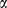 changes while 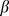 remains fixed, the distribution of 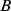 does not change, and as 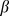 changes while 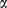 remains fixed, the distribution of 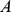 does not change. Note that the distribution of 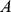 may but does not have to change in response to changes in 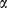, and analogously for 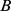 and 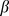.

Our approach is maximally general in the sense of applying to arbitrary sets of inputs and outputs (see Section 5 of Theory). To demonstrate it by detailed computations, however, we focus primarily on binary 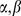 influencing binary 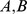; and even more narrowly, on the “homogeneous” case with the two values of both 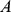 and 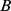 equiprobable at all values of the inputs 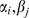 (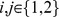),
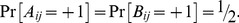
(7)Marginal selectivity then is satisfied trivially (because all marginal distributions are fixed).

**Figure 1:**
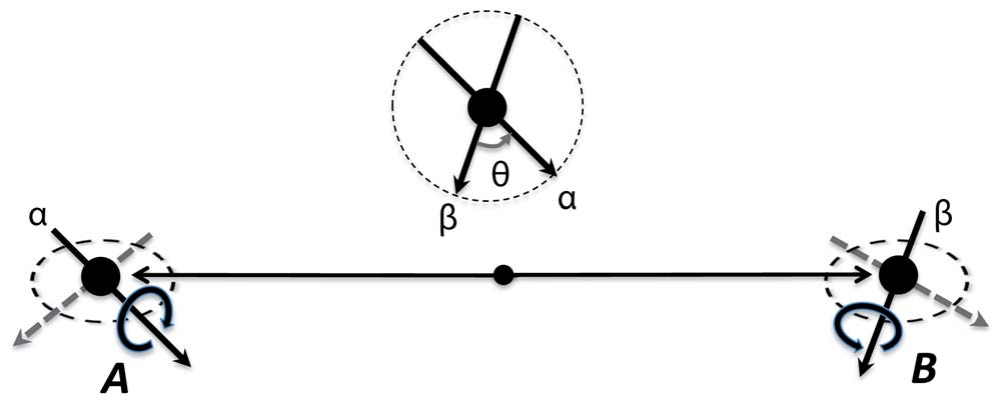
Entanglement paradigm. Schematic representation of two spin-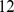 particles, e.g., electrons, in the singlet state (represented by 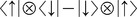 in quantum-mechanical notation) running away from each other. The directions 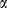 and 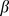 are detector settings for spin measurements (in our language, inputs). The measured spins 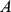 and 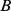 (outputs) in these directions are shown by rotation arrows: one direction of rotation (say, clockwise) represents “spin-up”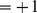 in one particle and “spin-down”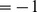 in the other. By the quantum theory, for any 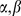, 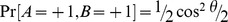 (equivalently, expected value of 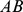 is 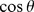). The two measurements are made simultaneously (in some inertial frame of reference).

The example focal for this paper is Bohm's version of the Einstein-Podolsky-Rosen paradigm (EPR/B) [9]: a quantum mechanical system consisting (in the simplest case) of two entangled spin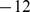 particles separated by a space-like interval (see Fig. 1). The two inputs here are spin measurements on these particles: input 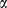 has two values corresponding to spin axes 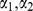 chosen for one particle, and input 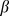 has two values corresponding to spin axes 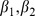 for another particle. The two outputs are spin values recorded: having chosen axes 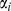 and 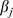, 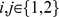, one records 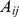 for the first particle and 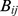 for the second, each being a random variable with values 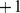 and 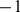. (Note that the spins of a given particle along two different axes are *noncommuting* (see Text S2), because of which if one spin value is determined precisely, +1 or −1, the other one has a nonzero uncertainty. This means that 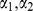 considered as measurements yielding precise values of spins are mutually exclusive, and this is the reason 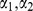 can be viewed as values of a single input 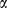; and analogously for 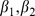 [10], [11].) Marginal selectivity (2) in this context is known under a variety of other names, such as “parameter independence” and “physical locality” [12]. We confine ourselves to the case (7), with the two spin values +1 and −1 being equiprobable for both 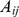 and 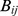.

Formally equivalent situations are abundant in behavioral and social sciences [8], [13]–[17], where the issue of selective influences was initially introduced in [18], [19], in the context of information processing architectures. An example of a system here (from our laboratory) can be a human observer who adjusts a visual stimulus until it matches in appearance another, “target” visual stimulus. Let the latter be characterized by two properties, 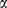 and 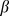 (e.g., amplitudes of two Fourier-components), each varying on two levels, 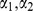 and 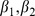. Denoting by 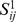 and 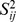 the corresponding properties (amplitudes) of the adjusted stimulus in response to 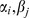, we define a binary random output 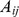 as having the value “high”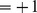 or “low”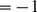 according as the variable 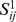 is above or below the median of its distribution; output 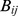 is defined from 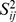 analogously. Marginal selectivity in the form (7) is ensured here by construction.

In an example from a biological domain 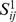 and 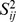 could be activity levels of two neurons tuned to two stimulus properties, 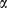 and 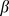, respectively. Making 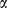 and 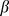 vary on two levels each and defining 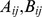 with respect to the medians of 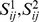 by the same rule as above, we get precisely the same mathematical formulation.

The formal equivalence of these three examples should by no means be interpreted as a hint at their physical affinity. Unlike in the EPR/Bohm paradigm, no physical laws prohibit the activity level 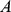 of a neuron tuned to stimulus property 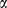 from being affected by stimulus property 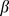. Similarly, the amplitude 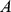 of the first Fourier component of the adjusted stimulus in the second example may very well be affected by the amplitude 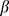 of the second Fourier component of the target stimulus. Our only claim is that if these “secondary” influences do not change the marginal distributions of 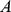 and 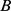 (which in the two examples in question is ensured by the definition of 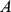 and 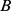), they can be viewed within the framework of a formal treatment that also includes the (physically very different) case of entangled particles.

## Theory

### 1 Forms of context (determinism)

In the following, symbols 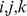 (possibly with primes) always take on values 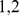 each, and each of the outputs 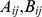 takes on values 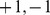 with equal probabilities. Representation (3) is equivalent to the existence of a jointly distributed system
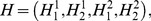
(8)such that every output pair 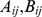 is distributed as 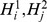; in symbols,
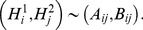
(9)As this entails
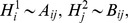
all components of 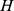 are random variables with equiprobable +1/−1, and (9) reduces to
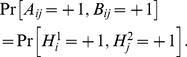
(10)The existence of 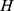 in (8) satisfying (9) is known as (a special case of) the *Joint Distribution Criterion* (JDC) [6], [7], [14], [20], [21]. It follows from (3) by
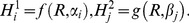
(11)Conversely, if (9) holds for some 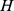, then one can put 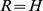 and
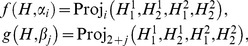
(12)where 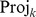 stands for the “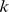 th member” (in the list of arguments). The JDC is a deep criterion that provides a probabilistic foundation for our understanding of the classical (non)contextuality (or classical determinism in physics). In particular, it immediately follows from the JDC that if representation (3) for 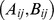 exists, the “hidden variables” 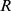 can always be reduced to a single discrete random variable with 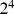 possible values (corresponding to the possible values of 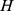).

Using the same notation as above,
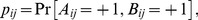
(13)the JDC in our case (two binary inputs and two binary outputs with equiprobable values) is equivalent to four double-inequalities
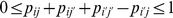
(14)with 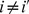, 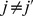 [6], [7]. (See Text S1 for a derivation.) They are often referred to as *the Bell/CHSH inequalities* (in the homogeneous form), CHSH acronymizing the authors of [4], although the first appearance of these inequalities dates to [5].

The theory of the EPR/B paradigm predicts and experimental data confirm violations of the Bell/CHSH inequalities [22], [23], but quantum mechanics imposes its own constraint on the same linear combinations of probabilities:
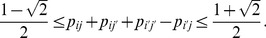
(15)This constraint is known as the Cirel'son inequalities [24], [25] (see Text S2 for a derivation). Since the class of vectors 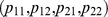 that satisfy these double-inequalities include those allowed by (14) as a proper subset, it is natural to expect that (15) represents some relaxation, or generalization of the JDC. No such generalization, however, has been previously proposed. Developing one is the main goal of this paper.

This generalization is not confined to quantum mechanical systems. In other (e.g., behavioral) applications, one cannot exclude a priori the possibility of the bounds 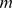 and 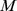 in
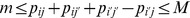
(16)being wider than in (15), or falling between the bounds in (14) and (15), or being more narrow than in (14). One can think of all kinds of other constraints imposed on the possible values of 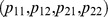, from confining this vector to one specific value to allowing it to vary freely. The latter (“complete chaos”) is represented by the “no-constraint” constraint
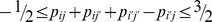
(17)with 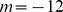 attained if one of 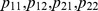 is 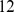 and the rest are zero, and 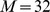 attained if three of 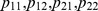 are 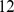 and the remaining one is zero. Recall that we only consider the outputs with equiprobable outcomes, so
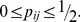
(18)All these conceivable constraints on the possible values of 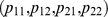 represent different forms and degrees of contextual influences. It would be unsatisfactory if all these possibilities, whether or not empirically realizable, could not be treated within a unified probabilistic framework including JDC as a special case. We construct such a framework, based on the classical (Kolmogorov's) theory of probability and the probabilistic coupling theory [26].

### 2 Connections

It is easy to see that for any vector of probabilities 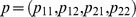 one can find a jointly distributed system of +1/−1 variables
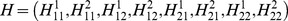
(19)such that
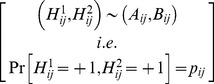
(20)for all 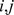. The JDC then amounts to additionally assuming that among all such vectors 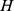 there is one with
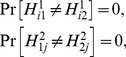
(21)and this is the assumption that is rejected by quantum theory in the EPR/B paradigm. Once (21) is explicitly formulated, however, it becomes clear that it is not the only way of thinking of 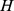. Since 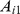 and 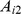 occur under mutually exclusive conditions, one cannot identify the distribution of 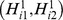 with that of 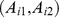. The latter does not exist as a pair of jointly distributed random variables. There is therefore no privileged pairing scheme for realizations of 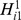 and 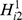,and zero values for 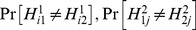 are as acceptable a priori as any other. Analogous considerations apply to 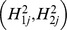 and 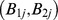.

Our approach consists in replacing (21) with more general
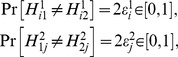
(22)and characterizing the dependence of 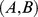 on 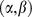 by properties of the set of all 4-vectors 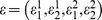 that are compatible with or imply certain constraints imposed on the vectors 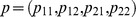. Having adopted a particular diagram of input-output correspondences (in our case, 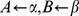), we can also say that these sets of 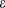 characterize the contextual role of 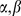 for 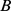 and 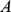, respectively.

We call 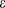 a vector of *connection probabilities*. The connection probabilities are of a principally non-empirical nature: they are joint probabilities of events that can never co-occur. By contrast, due to (20) the components of 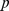 are joint probabilities of events that do co-occur, and by observing these co-occurrences the probabilities in 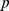 can be estimated. To emphasize this distinction we refer to 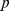 as a vector of *empirical probabilities*.

To distinguish our approach from other forms and meanings of probabilistic contextualism, e.g., [27], [28], [29], we dub it the “*all-possible-couplings”* approach. The term “coupling” refers to imposing a joint distribution (say, that of 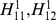) on random variables that otherwise are not jointly distributed (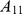 and 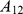). For a rigorous and general discussion of couplings and connections see Section 5.

### 3 Extended Linear Feasibility Polytope (ELFP)

ELFP is the set of all possible 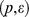 for which there exists a vector 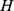 in (19) with jointly distributed components 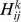 such that (20) holds, and, in accordance with (22),
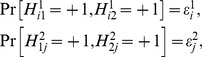
(23)for all 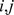. The existence of such an 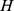 means the existence of a probability vector 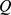 consisting of the 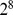 joint probabilities
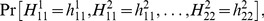
(24)

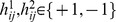
. Let 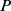 denote the 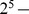
component vector consisting of 2^4^ empirical probabilities
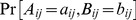
(25)and 2^4^ connection probabilities
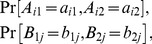
(26)

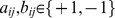
.

Define a 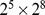 Boolean matrix 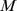 whose rows are enumerated in accordance with components of 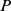 (i.e., by equalities 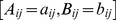, 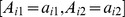, or 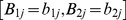) and columns in accordance with components of 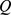 (i.e., by equalities 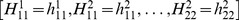). An entry of 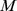 contains 1 if and only if the corresponding random variables in the enumerations of its row and its column have the same values: e.g., if a row is enumerated by 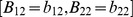 and a column by 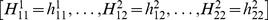, then their intersection contains 1 if and only if 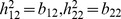.

It is easy to see that 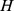 exists if and only if
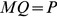
(27)for some vector 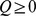 (componentwise) of probabilities. The vectors 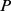 for which such a 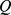 exists are exactly those within the polytope whose vertices are the columns of the matrix 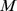. The term ELFP is due to this construction extending that of the linear feasibility test in [10]. This test, among other applications, is the most general way of extending the Bell/CHSH criterion to an arbitrary number of particles, spin axes, and spin quantum numbers [10], [11], [30]–[32]. Its application to binary inputs/outputs (not necessarily with equiprobable outcomes) is shown in Text S1.

To describe ELFP by inequalities on 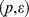, we introduce the 16-component sets
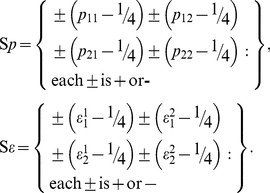
(28)

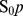
 and 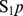 denote the subsets of 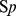 with, respectively, even (0,2, or 4) and odd (1 or 3) number of 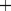 signs; 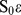 and 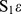 are defined analogously. ELFP is described by
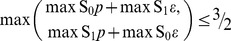
(29)(see Text S3).

### 4 All, Fit, Force, and Equi sets

Let 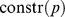 denote any constraint (e.g., inequalities) imposed on 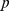. Our approach consists in characterizing this constraint by solving the following four problems:

1. Find the set 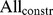 of all 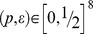 with 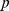 subject to 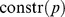: i.e., 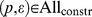 if and only if
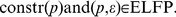
(30)
2. Find the set 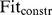 of connection vectors 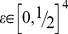 that fit (are compatible with) all empirical probability vectors 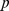 satisfying 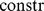: i.e., 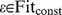 if and only if
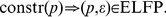
(31)
3. Find the set 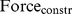 of 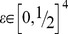 that force all compatible empirical probability vectors 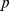 to satisfy 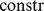: i.e., 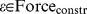 if and only if
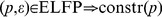
(32)
4. Find the set 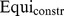 of 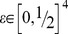 for which an empirical probability vector 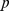 satisfies 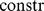 if and only if 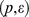 is in the ELFP set: i.e., 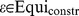 if and only if
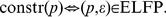
(33)Clearly, 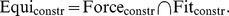

To illustrate, we focus on the following four benchmark constraints. The no-constraint, or “complete chaos” situation is given by
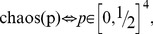
(34)equivalent to (17). The quantum mechanical constraint is given by
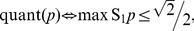
(35)equivalent to (15). The “classical” constraint is given by
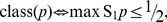
(36)equivalent to the Bell/CHSH inequalities (14). Finally, we consider the constraint
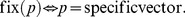
(37)For all constraints except for 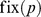 the sets All, Fit, Force, and Equi are as shown in Table 1 (for derivations see Text S4).

**Table 1:** Characterizations of the sets of four different types (columns) subject to three constrains (rows). In all cells, 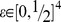 and 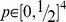.

	All 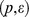	Fit 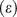	Force 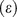	Equi 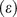
chaos	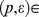 ELFP	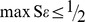	arbitrary	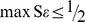
quant	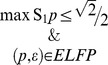	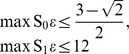	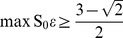	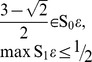
class	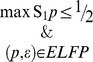	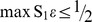	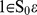	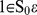

Thus, 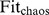 is the set of all 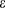 such that 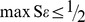: if an 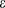 is in this set, then any 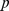 (with no constraints) is compatible with it. 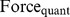 is characterized by 
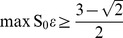
: if an 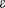 is in this set, then all compatible with it 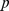 satisfy 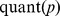. 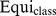 is the set of all 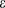 such that 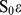 contains 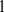: for any such an 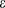, a 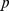 is compatible with it if and only if it satisfies .

**Figure 2:**
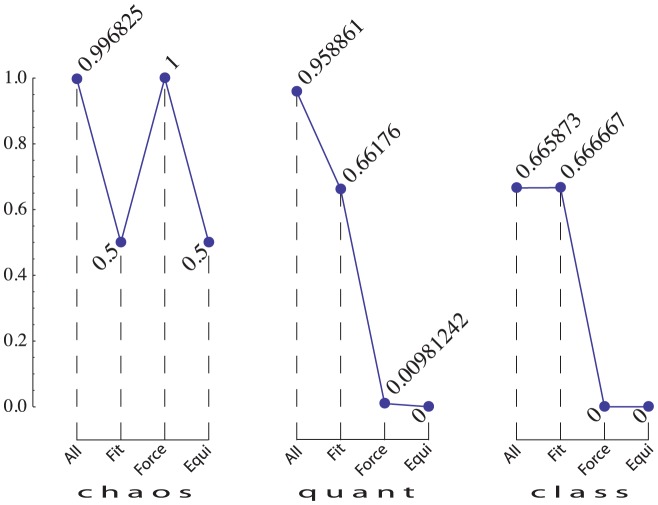
Volume profiles under constraints. Profiles 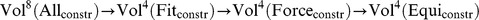 for constraints chaos, quant, and class.

For each of these sets we compute 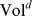, its volume normalized by that of 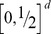, with 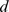 being the dimensionality of the set (Fig. 2). Thus, the defining property of 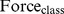, 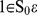, is satisfied if and only if either all 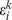 are 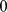, or they all are 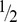, or two of them are 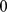 and two 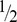. Hence 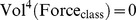. For nonzero volumes, the derivation is described in Text S4. Each panel of Fig. 2 can be viewed as a “profile” of the corresponding constraint. Each of the first three volumes in a panel can be viewed as characterizing the “strictness” of a constraint, in three different meanings. The intuition of a stricter constraint is that it corresponds to a smaller 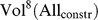, larger 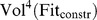, and smaller 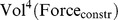. Characterizing constraints imposed on empirical probabilities by multidimensional volumes is not a new idea [33], but our computations are different: they are aimed at sets of nonempirical connection probabilities in relation to constraints imposed on empirical probabilities.

**Figure 3:**
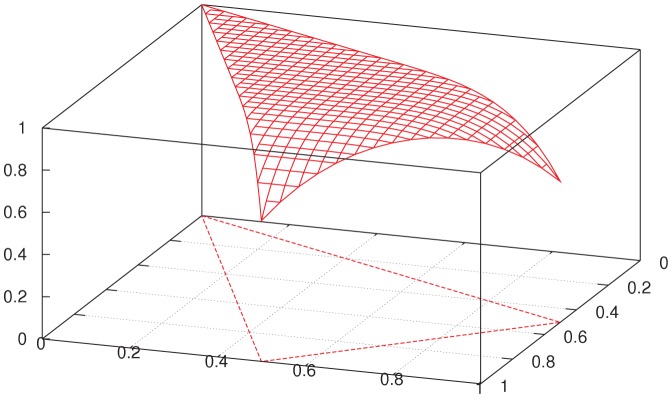
Fit-set volumes for fixed probabilities. 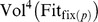
 is shown as a function of 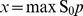 and 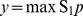. The possible 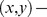
pairs form the triangle 
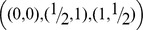
, and 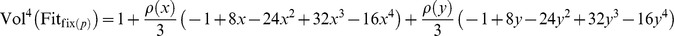, where 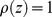 if 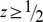 and 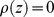 otherwise.

The constraint 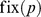 has to be handled separately. Clearly, 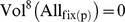. 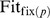 is described by
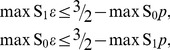
(38)and 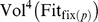 is a polynomial function of 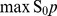 and 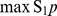, these two quantities forming the triangle 
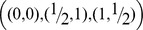
. The polynomial and its values are shown in Fig. 3 (see Text S5, for computational details). 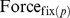 is clearly empty, hence so is 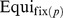.

### 5 All-possible-couplings approach on the general level

We show here how the approach presented so far generalizes to arbitrary sets of inputs and random outputs. We use the term *sequence* to refer to any indexed family (a function from an index set into a set), with index sets not necessarily countable. We present sequences in the form 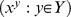, 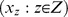, or 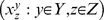. A random variable is understood most broadly, as a measurable mapping between any two probability spaces. In particular, any sequence of jointly distributed random variables is a random variable. For brevity, we omit an explicit presentation of probability spaces and distributions. In all other respects the notation and terminology closely follow [15], [11].

An *input* is a set of elements called *input values*. Let 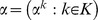 be a sequence of inputs. A *treatment* is a sequence 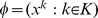 that belongs to a nonempty set 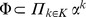 (so that 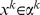 for all 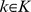). If 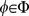, 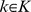, and 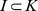, then 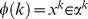 and 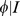 is the restriction of 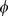 to 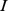, i.e., the sequence 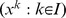.

An *output* is a random variable. Let 
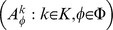
 be a sequence of outputs such that

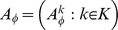
 is a random variable for every 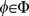, i.e., the random variables 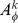 across all possible 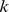 possess a joint distribution;
2. if 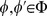, 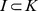, and 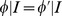, then 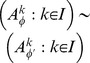.

Property 2 is *(complete) marginal selectivity* [8]. 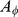 is called an *empirical random variable*, and 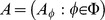 is the *sequence of empirical random variables*.

*Remark* 1. The interpretation is that for every 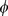, each 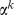 may “directly” influence 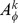 but no other output in 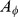. The fact that inputs in 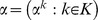 and outputs in an empirical random variable 
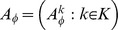
 are in a bijective correspondence is not restrictive: this can always be achieved by an appropriate grouping of inputs and (re)definition of treatments 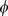 [10].

*Remark* 2. The special case considered in the previous sections corresponds to 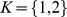,
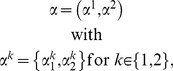
(39)

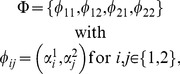
(40)and (abbreviating 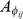 as 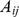 and 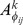 as 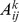)
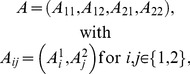
(41)where each 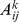 is a binary random variable with 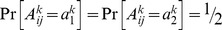.

Given a sequence of *empirical random variables* 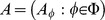, a sequence of random variables
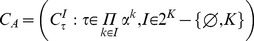
(42)(not necessarily jointly distributed) is called a *connecting set* for 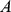 if each 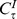 is a coupling for
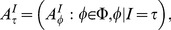
(43)where 
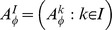
. This means that 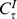 is a random variable of the form
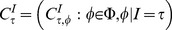
(44)with
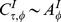
(45)for all 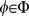 such that 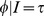. 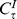 is called an 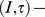 *connection*. The indexation in 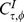 is to ensure that if 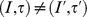, then 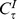 and 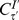 are stochastically unrelated. An *identity* 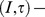 *connection* 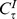 is one with 
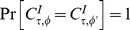
 for any 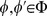.

*Remark* 3. It is generally convenient not to distinguish identically distributed connections. By abuse of language, the distribution of 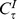 (or some characterization thereof) can also be called 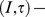
connection. We used this language in the previous sections when we represented 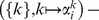
connections (without introducing them explicitly) by probabilities 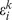 and called 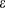 a connection vector. See Remark 4.

A jointly distributed sequence
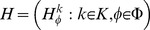
(46)is called an *Extended Joint Distribution Sequence* (EJDS) for 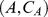 if for any 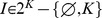 and any 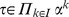,
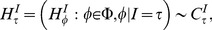
(47)where 
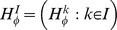
, and
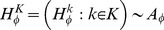
(48)for any 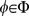.

*Remark* 4. For the special case considered in the previous sections, a connecting set for 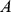 is (conveniently replacing 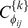, 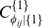, and 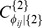 with 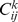, 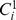, and 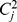, respectively)
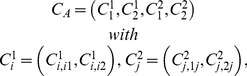
(49)such that
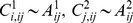
(50)for 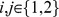. An EJDS for 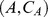 is a random variable (using analogous abbreviations)
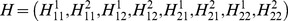
(51)such that
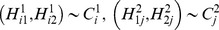
(52)and
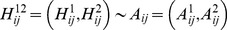
(53)for 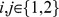. In the previous sections each 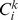 was represented by 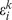 and each 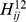 by 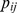.

An EJDS for 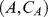 reduces to the Joint Distribution Criterion set (JDC set) of the theory of selective influences [11], [14] if all connections in 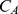 are identity ones. Note that no connection has an empirical meaning: for distinct 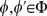, the variables 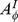 and 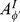 corresponding to 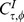 and 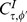 do not have an empirically observable (or theoretically privileged) pairing scheme.

Let 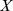 be any set whose elements are sequences of *empirical random variables* 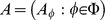. 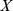 can be viewed as the set of all possible *empirical random variables* satisfying certain constraints. We define the sets All*_X_*, Fit*_X_*, Force*_X_*, and Equi*_X_* as follows:

1. All*_X_* is the set of all pairs 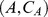 such that
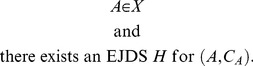
(54)
2. Fit*_X_* is the set of all 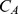 such that
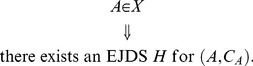
(55)
3. Force*_X_* is the set of all 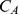 such that
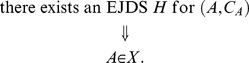
(56)

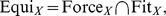
 that is, 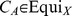 if and only if
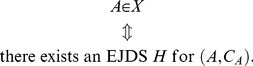
(57)

The all-possible-couplings approach in the general case consists in characterizing any 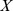 (interpreted as a type of contextuality or determinism) by All*_X_*, Fit*_X_*, Force*_X_*, and Equi*_X_*. A straightforward generalization of this approach that might be useful in some applications is to replace 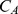 in all definitions with a subset of 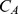, or several subsets of 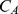 tried in turn. Thus one might consider connections involving only particular 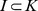 (e.g., only singletons), or one might require that some of the connections are identity ones.

## Conclusion

The essence of the proposed mathematical framework is as follows. We consider all possible couplings for empirically observed vectors of random outputs. In the case of two binary inputs/outputs these vectors are pairs
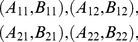
(58)the couplings 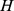 for them have the form (19), with the coupling relation (20). We assume that the joint distributions (in our case described by pairwise joint probabilities) of the empirically observed 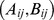 are subject to a certain constraint, given to us by substantive considerations outside the scope of our approach: for instance, if a system consists of entangled particles, a constraint, say (15), is derived from the quantum theory. Due to (20), the constraint is imposed on
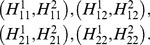
(59)We investigate then the unobservable “connections”, the subvectors of the components of 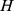 that correspond to outputs obtained at mutually exclusive values of the inputs (i.e., never co-occurring). In our case these are the pairs
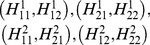
(60)corresponding to, respectively,
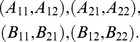
(61)We then characterize the constraint imposed on the empirical pairs (59) by describing the “fitting” or “forcing” (or both “fitting and forcing”) distributions of the unobservable connections (60). By fitting distributions of (60) we mean those that are compatible with any (59) subject to the constraint in question, the compatibility meaning that all these eight pairs can be embedded into a single 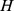 (with jointly distributed components). By forcing distributions of (60) we mean those that are compatible with (59) only if the latter are subject to the given constraint.

The value of this approach is in providing a unified language for speaking of probabilistic contextuality. At the cost of greater computational complexity but with no conceptual complications the computations involved in our demonstration of the all-possible-couplings approach can be extended to more general cases: arbitrary marginal probabilities (satisfying marginal selectivity), nonlinear constraints, and greater numbers of inputs, outputs, and their possible values. The language for a completely general theory, involving unrestricted (not necessarily finite) sets of inputs, outputs, and their values, is presented in Section 5 of Theory.

## Supporting Information

Text S1 **Derivation of the Bell/CHSH bounds.**
(PDF)

Text S2 **Derivation of the Cirel'son bounds.**
(PDF)

Text S3 **Computations for ELFP.**
(PDF)

Text S4
**Computations for** 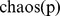 **,** 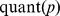 **, and** 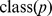 **constraints.**
(PDF)

Text S5
**Computations for** 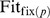 **constraint.**
(PDF)
