# Supplementary material for: All-Possible-Couplings Approach to Measuring Probabilistic Context
Source: PLoS One. 2013 May 6;8(5):e61712. doi: 10.1371/journal.pone.0061712 (PMC3646012; doi:10.1371/journal.pone.0061712)
Supplement: Text S1 — Derivation of the Bell/CHSH bounds. (PDF) [file pone.0061712.s001.pdf]

S1 Derivation of the Bell/CHSH bounds

A representation (8)-(9) exists if and only if the 2^4 possible values $\left(h_1^1, h_2^1, h_1^2, h_2^2\right)$ of H ($h_i^k \in \{+1, -1\}, i, k \in \{1, 2\}$) can be assigned probabilities

$$p\left(h_1^1, h_2^1, h_1^2, h_2^2\right) = \Pr\left[H_1^1 = h_1^1, H_2^1 = h_2^1, H_1^2 = h_1^2, H_2^2 = h_2^2\right], \tag{S1.1}$$

so that, for all $a_{ij}, b_{ij} \in \{+1, -1\}$, $i, j \in \{1, 2\}$,

$$\sum_{h_1^1, h_2^1, h_1^2, h_2^2} \chi\left(h_i^1 = a_{ij} \wedge h_j^2 = b_{ij}\right) p\left(h_1^1, h_2^1, h_1^2, h_2^2\right) = \Pr\left[A_{ij} = a_{ij}, B_{ij} = b_{ij}\right], \tag{S1.2}$$

where $\chi(...)$ indicates the truth value (1 or 0) of the statement within the parentheses. It is easy to see that this system of linear equations can be written as

$$MQ = P, \tag{S1.3}$$

where P is the 16-vector of probabilities $\Pr\left[A_{ij} = a_{ij}, B_{ij} = b_{ij}\right]$ indexed (together with the columns of matrix M) by (i, j, a_{ij}, b_{ij})-values, say, lexicographically; Q is the 16-vector of unknown probabilities $p\left(h_1^1, h_2^1, h_1^2, h_2^2\right)$ indexed (together with the rows of M) by $\left(h_1^1, h_2^1, h_1^2, h_2^2\right)$-values in some order; and the cells of M indexed by $\left((i, j, a_{ij}, b_{ij}), \left(h_1^1, h_2^1, h_1^2, h_2^2\right)\right)$ contain $\chi\left(h_i^1 = a_{ij} \wedge h_j^2 = b_{ij}\right)$. We conclude that a representation (8)-(9) exists if and only if

$$B(M, P) = 1, \tag{S1.4}$$

where $B(M, P)$ is a Boolean function equal to 1 if (**??**) has at least one solution with nonnegative components of Q. It is easy to show (see [10] for details) that solutions Q of (**??**) always have the property

$$\sum_{h_1^1, h_2^1, h_1^2, h_2^2} p\left(h_1^1, h_2^1, h_1^2, h_2^2\right) = 1. \tag{S1.5}$$

It is known from the linear programming theory that $B(M, P)$ is always computable. A standard facet enumeration algorithm allows one to obtain the system of all linear inequalities and equations imposed on P that are equivalent to (**??**). This system turns out to consist of the equalities (2) representing marginal selectivity, and inequalities that can be written as

$$-2 \leq \mathrm{E}_{ij} + \mathrm{E}_{i'j} + \mathrm{E}_{i'j'} - \mathrm{E}_{ij'} \leq 2, \tag{S1.6}$$

where, in reference to (1), $\mathrm{E}_{ij} = p_{ij} + s_{ij} - q_{ij} - r_{ij}$ is the expected value of $A_{ij}B_{ij}$. When marginal probabilities are all $1/2$, these inequalities reduce to (14), using $p_{ij} = (\mathrm{E}_{ij} + 1)/4$.

Remark 1. It would be a mistake to consider this proof "computer-assisted" because it mentions a facet enumeration algorithm. The latter is merely a long chain of trivial algebraic transformations, that can always be written out *in extenso* if needed.
