# Supplementary material for: All-Possible-Couplings Approach to Measuring Probabilistic Context
Source: PLoS One. 2013 May 6;8(5):e61712. doi: 10.1371/journal.pone.0061712 (PMC3646012; doi:10.1371/journal.pone.0061712)
Supplement: Text S2 — Derivation of the Cirel'son bounds. (PDF) [file pone.0061712.s002.pdf]

S2 Derivation of the Cirel'son bounds

The following is a modification of the derivation given in [25]. Let a, a', b, b' be the Hermitian operators in complex Hilbert space corresponding to, respectively, outputs $A_{1j}, A_{2j}, B_{i1}, B_{i2}$ (where i and j are irrelevant, i.e., a represents both A_{11} and A_{12}, b both B_{11} and B_{21}, etc.). Denoting by E expected value and by Tr trace, we have, for any state (density operator) W,

$$\begin{array}{c} 4p_{11} - 1 = \mathrm{E}\left[A_{11}B_{11}\right] = \mathrm{Tr}\left(Wab\right), \\ 4p_{12} - 1 = \mathrm{E}\left[A_{12}B_{12}\right] = \mathrm{Tr}\left(Wab'\right), \\ etc. \end{array} \tag{S2.1}$$

where either of a and a' commutes with either of b and b'. Inequalities (15) to be demonstrated are equivalent to

$$\begin{array}{c} R_1 = \left|\mathrm{Tr}\left(Wab\right) + \mathrm{Tr}\left(Wab'\right) + \mathrm{Tr}\left(Wa'b\right) - \mathrm{Tr}\left(Wa'b'\right)\right| = \left|\mathrm{Tr}\left(Ws_1\right)\right| \leq 2\sqrt{2}, \\ R_2 = \left|\mathrm{Tr}\left(Wab\right) + \mathrm{Tr}\left(Wab'\right) - \mathrm{Tr}\left(Wa'b\right) + \mathrm{Tr}\left(Wa'b'\right)\right| = \left|\mathrm{Tr}\left(Ws_2\right)\right| \leq 2\sqrt{2}, \\ etc. \end{array} \tag{S2.2}$$

where

$$\begin{array}{c} s_1 = ab + ab' + a'b - a'b' = a\left(b + b'\right) + a'\left(b - b'\right), \\ s_2 = ab + ab' - a'b + a'b' = a\left(b + b'\right) - a'\left(b - b'\right), \\ etc. \end{array} \tag{S2.3}$$

Since the values of the outputs, +1/-1, are the eigenvalues of the corresponding operators, it can easily be seen (e.g., by spectral decomposition, squaring, and then multiplication by an arbitrary vector) that

$$a^2 = b^2 = a'^2 = b'^2 = I, \tag{S2.4}$$

where I is the identity operator. Using this we show by straightforward if somewhat tedious algebra that

$$\begin{array}{c} s_1^2 = s_4^2 = 4I - \left(aa' - a'a\right)\left(bb' - b'b\right), \\ s_2^2 = s_3^2 = 4I + \left(aa' - a'a\right)\left(bb' - b'b\right), \end{array} \tag{S2.5}$$

whence, using the conventional notation for commutators, $[x, y] = xy - yx$,

$$\begin{array}{c} \mathrm{Tr}\left(Ws_1^2\right) = \mathrm{Tr}\left(Ws_4^2\right) = 4 - \mathrm{Tr}\left(W\left[a, a'\right]\left[b, b'\right]\right), \\ \mathrm{Tr}\left(Ws_2^2\right) = \mathrm{Tr}\left(Ws_3^2\right) = 4 + \mathrm{Tr}\left(W\left[a, a'\right]\left[b, b'\right]\right). \end{array} \tag{S2.6}$$

For $k = 1, 2, 3, 4$, since s_k is a Hermitian operator (as the sum of products of commuting Hermitian operators), we know that

$$0 \leq \left(\mathrm{Tr}\left(Ws_k\right)\right)^2 \leq \mathrm{Tr}\left(Ws_k^2\right). \tag{S2.7}$$

It follows from (S2.6) then that

$$\left|\mathrm{Tr}\left(W\left[a, a'\right]\left[b, b'\right]\right)\right| \leq 4 \tag{S2.8}$$

and

$$\mathrm{Tr}\left(Ws_k^2\right) \leq 8. \tag{S2.9}$$

But then

$$R_k^2 = \left(\mathrm{Tr}\left(Ws_k\right)\right)^2 \leq 8. \tag{S2.10}$$

This implies (S2.2) and (15).

That the value $2\sqrt{2}$ in (S2.2) can be attained is easy to show using the EPR/B paradigm: if $\alpha_1 = 0$, $\alpha_2 = \pi/2$, $\beta_1 = \pi/4$, $\beta_2 = -\pi/4$, then

$$R_1 = \cos\left(\alpha_1 - \beta_1\right) + \cos\left(\alpha_1 - \beta_2\right) + \cos\left(\alpha_2 - \beta_1\right) - \cos\left(\alpha_2 - \beta_2\right) = 2\sqrt{2}. \tag{S2.11}$$

Remark 1. It is instructive to see that if the operators a, a' (or b, b') commute, (S2.6) leads to $R_k^2 \leq 4$, which, in view of (S2.1), is equivalent to (14). It is tempting therefore to consider (14) as merely a special (commutative) case of the construction used above to prove (15). Notice however that this view cannot be accepted without additional arguments: the proof of (14) makes no use of the assumption that the outputs are eigenvalues of Hermitian operators in a Hilbert space.

Remark 2. It is known from [6, 7] that if a vector $(p_{11}, p_{12}, p_{21}, p_{22})$ satisfies (14), then this vector can be generated by a system with binary inputs and equiprobable binary outputs that satisfies (3), that is, is explainable by classical (non)contextuality. By contrast, if a vector $(p_{11}, p_{12}, p_{21}, p_{22})$ satisfies (15), it is not known whether this vector can be generated by a quantum mechanical system with binary inputs and equiprobable binary outputs. In this sense our characterization of quantum contextuality is improvable.
