# Supplementary material for: All-Possible-Couplings Approach to Measuring Probabilistic Context
Source: PLoS One. 2013 May 6;8(5):e61712. doi: 10.1371/journal.pone.0061712 (PMC3646012; doi:10.1371/journal.pone.0061712)
Supplement: Text S3 — Computations for ELFP. (PDF) [file pone.0061712.s003.pdf]

S3 Computations for ELFP

A convex bounded polytope can be equivalently defined either as the convex hull of a set of points (V-representation) or as the intersection of half-spaces (H-representation). For our purposes, a V-representation of a convex polytope in d-space is given by a set of points $x_1, \ldots, x_n \in \mathbb{R}^d$. The polytope consists of all convex combinations of these points: $\lambda_1 x_1 + \cdots + \lambda_n x_n$, for all $\lambda_1, \ldots, \lambda_n \geq 0$, $\lambda_1 + \cdots + \lambda_n = 1$. It is possible that the polytope is of lower dimension than the space $\mathbb{R}^d$ in which it is defined if all the points x_i reside in a lower dimensional affine subspace of $\mathbb{R}^d$. A minimal V-representation (including only extreme points, i.e., points that are vertices of the polytope) is unique. The H-representation of a convex polytope is given by vectors $a_1, \ldots, a_m \in \mathbb{R}^d$ and a vector $b \in \mathbb{R}^m$. The polytope consists of the points $x \in \mathbb{R}^d$ satisfying $a_i^T x \leq b_i$ for all $i = 1, \ldots, m$. A lower-dimensional convex polytope can be represented by including inequalities of the forms $a^T x \leq b$ and $(-a)^T x \leq -b$ for some a and b or by explicitly specifying certain constraints as equations in the representation. For a full-dimensional convex polytope, the minimal H-representation is unique. However, for a lower-dimensional polytope, the equation constraints can be specified in many equivalent ways and the inequality constraints can look different depending on which of the linearly related coordinates are used to specify them.

There exist algorithms for converting between the two representations of a convex polytope in exact rational arithmetic. We have used our own program for these conversions but other programs, such as *lrs* (http://cgm.cs.mcgill.ca/~avis/C/lrs.html), can do the same. The conversion between the two representation is computationally demanding, the algorithms generally requiring superpolynomial time in the size of the input.

A computationally simpler problem is eliminating redundant points (those that are not vertices of the polytope) from a V-representation or eliminating redundant equations or inequalities from an H-representation. This problem can be solved by linear programming and the algorithm is implemented in the *redund* program that comes with *lrs*. However, the *redund* program is not sufficient for putting an H-representation to a minimal form as it cannot convert sets of inequalities into equivalent equations (e.g., the three inequalities $x \geq 0$, $y \geq 0$, $x + y \leq 0$ should be minimally represented as the two equations $x = 0$, $y = 0$). To find the minimal H-representation, for every constraint $a_i^T x \leq b_i$ or $a_i^T x = b_i$ in turn, one can find the upper and lower bounds u and l by maximizing and minimizing the expression $a_i^T x$ given the other constraints, and apply the following rules:

1. if this is an equation constraint (i.e., $a_i^T x = b_i$) and $u = l = b_i$, then the constraint is redundant and can be eliminated;

2. if this is an inequality constraint (i.e., $a_i^T x \leq b_i$) and $u \leq b_i$, then the inequality is redundant and can be eliminated. Otherwise, if $l = b_i$, then the constraint should be converted to an equation.

The dimension of a polytope can be determined from a minimal H-representation. It is the dimension of the space minus the number of equation constraints in the minimal representation. Given a full-dimensional polytope, its volume can be computed using the *lrs* program alongside the conversion from a V-representation to an H-representation. If the polytope is given as an H-representation, then it has to be converted to a V-representation first to compute its volume using *lrs*. To compute the volume of a lower-dimensional polytope, we first move to a lower-dimensional parameterization that spans the affine subspace where the polytope resides.

To compute ELFP, we begin by formulating the linear programming problem $MQ = P$ subject to $Q \geq 0$, as described in the main text (M being $2^5 \times 2^8$, P having 2^5 components). M defines the V-representation for ELFP, and Vol^8 for ELFP is computed directly from it. Applying an algorithm to find an equivalent H-representation we obtain a system of 160 inequalities and 16 equations. We can then substitute the expressions in the above matrices into this system and reduce any redundant inequalities and equations. The resulting system has 144 nonredundant inequalities and no equations with the $p_{11}, p_{12}, p_{21}, p_{22}, \varepsilon_1^1, \varepsilon_2^1, \varepsilon_1^2, \varepsilon_2^2$ variables. Then (dropping the implicit $\varepsilon \in [0, 1/2]^4$ and $p \in [0, 1/2]^4$

constraints), we algebraically simplify the list of 144 inequalities, first into

$$\begin{aligned} -\Gamma \leq -p_{11} + p_{21} + p_{12} + p_{22} &\leq 1 + \Gamma, \\ -\Gamma \leq p_{11} - p_{21} + p_{12} + p_{22} &\leq 1 + \Gamma, \\ -\Gamma \leq p_{11} + p_{21} - p_{12} + p_{22} &\leq 1 + \Gamma, \\ -\Gamma \leq p_{11} + p_{21} + p_{12} - p_{22} &\leq 1 + \Gamma, \end{aligned} \tag{S3.1}$$

$$-\Lambda \leq p_{11} + p_{21} + p_{12} + p_{22} \leq 2 + \Lambda, \tag{S3.2}$$

$$\begin{aligned} |-p_{11} - p_{21} + p_{12} + p_{22}| &\leq 1 + \Lambda, \\ |-p_{11} + p_{21} - p_{12} + p_{22}| &\leq 1 + \Lambda, \\ |-p_{11} + p_{21} + p_{12} - p_{22}| &\leq 1 + \Lambda, \end{aligned} \tag{S3.3}$$

where

$$\begin{aligned} \Gamma = \min\{ \quad & 1 - \varepsilon_1^1 - \varepsilon_1^2 + \varepsilon_2^1 + \varepsilon_2^2, \\ & 1 - \varepsilon_1^1 + \varepsilon_1^2 - \varepsilon_2^1 + \varepsilon_2^2, \\ & 1 - \varepsilon_1^1 + \varepsilon_1^2 + \varepsilon_2^1 - \varepsilon_2^2, \\ & 1 + \varepsilon_1^1 - \varepsilon_1^2 - \varepsilon_2^1 + \varepsilon_2^2, \\ & 1 + \varepsilon_1^1 - \varepsilon_1^2 + \varepsilon_2^1 - \varepsilon_2^2, \\ & 1 + \varepsilon_1^1 + \varepsilon_1^2 - \varepsilon_2^1 - \varepsilon_2^2, \\ & \varepsilon_1^1 + \varepsilon_1^2 + \varepsilon_2^1 + \varepsilon_2^2, \\ & 2 - \varepsilon_1^1 - \varepsilon_1^2 - \varepsilon_2^1 - \varepsilon_2^2 \}, \end{aligned} \tag{S3.4}$$

$$\begin{aligned} \Lambda = \min\{ - \quad & \varepsilon_1^1 + \varepsilon_1^2 + \varepsilon_2^1 + \varepsilon_2^2, \\ & \varepsilon_1^1 - \varepsilon_1^2 + \varepsilon_2^1 + \varepsilon_2^2, \\ & \varepsilon_1^1 + \varepsilon_1^2 - \varepsilon_2^1 + \varepsilon_2^2, \\ & \varepsilon_1^1 + \varepsilon_1^2 + \varepsilon_2^1 - \varepsilon_2^2, \\ & 1 - \varepsilon_1^1 - \varepsilon_1^2 - \varepsilon_2^1 + \varepsilon_2^2, \\ & 1 - \varepsilon_1^1 - \varepsilon_1^2 + \varepsilon_2^1 - \varepsilon_2^2, \\ & 1 - \varepsilon_1^1 + \varepsilon_1^2 - \varepsilon_2^1 - \varepsilon_2^2, \\ & 1 + \varepsilon_1^1 - \varepsilon_1^2 - \varepsilon_2^1 - \varepsilon_2^2 \}, \end{aligned} \tag{S3.5}$$

and then, by noticing regularities, into the compact inequality (29).

Remark 1. Changing $\varepsilon_j^i \to 1/2 - \varepsilon_j^i$ leads to (denoting the new ε-vector by ε')

$$\max \mathsf{S}_1\varepsilon' = \max \mathsf{S}_0\varepsilon, \max \mathsf{S}_0\varepsilon' = \max \mathsf{S}_1\varepsilon. \tag{S3.6}$$

Analogously for $p_{ij} \to 1/2 - p_{ij}$,

$$\max \mathsf{S}_1 p' = \max \mathsf{S}_0 p, \max \mathsf{S}_0 p' = \max \mathsf{S}_1 p. \tag{S3.7}$$

It follows that we cannot without loss of generality confine all components of ε or p to $[0, 1/4]$. But ELFP does not change if the transformation $x \to 1/2 - x$ is applied to an even number of the components of (p, ε).
