# Supplementary material for: All-Possible-Couplings Approach to Measuring Probabilistic Context
Source: PLoS One. 2013 May 6;8(5):e61712. doi: 10.1371/journal.pone.0061712 (PMC3646012; doi:10.1371/journal.pone.0061712)
Supplement: Text S4 — Computations for , , and constraints. (PDF) [file pone.0061712.s004.pdf]

S4 Computations for chaos(p), quant(p), and class(p) constraints

The $\text{All}_{\text{constr}}$ polytopes for the three constraints are obtained by concatenating the ELFP equations and inequalities with the constraint inequalities. Then, the volumes are computed by using the *lrs* program as described above.

For $\text{Fit}_{\text{constr}}$ polytopes, we observe first that they are convex. This follows from

$$\begin{aligned}\text{Fit}_{\text{constr}} &= \{\varepsilon : \forall i = 1, \ldots, n : (p_{(i)}, \varepsilon) \in \text{ELFP}\} \\ &= \text{ELFP}_{p_{(1)}} \cap \cdots \cap \text{ELFP}_{p_{(n)}},\end{aligned} \tag{S4.1}$$

where $p_{(i)}$, $i = 1, \ldots, n$, denote the vertices of the 4D convex polytope defined by constr and $\text{ELFP}_{p_{(i)}}$ denotes the (convex) cross-section of the ELFP set formed with $p = p_{(i)}$. It follows that $\text{Fit}_{\text{constr}}$ is convex as the intersection of convex sets. Following the logic of this observation, we have implemented a general program for eliminating variables from a system of linear equations and inequalities so that the resulting system is satisfied for exactly those values for which there exist such values of the eliminated variables for which the original system is satisfied. This program together with steps to ensure that the resulting representation is minimal was used to find all the Fit sets shown in the main text.

Finding the forcing sets is more difficult as they are generally not convex. We characterize them using the equation

$$\begin{aligned}&\text{Force}_{\text{chaos}} - \text{Force}_{\text{constr}} \\ &= \{\varepsilon : (\exists p : (p, \varepsilon) \in \text{ELFP} \wedge \neg\text{constr}\,(p))\}\,.\end{aligned} \tag{S4.2}$$

This equation provides an algorithm: for each inequality in constr, form the conjunction of the ELFP inequalities with the negation of the inequality. Then project this conjunction to the ε 4-space. The union of these projections over all inequalities in constr is the set $\text{Force}_{\text{chaos}} - \text{Force}_{\text{constr}}$. We have implemented a general program that takes as input a representation of a polytope, a list of additional constraints, and a list of variables to eliminate. It then outputs a representation of the difference of the polytope and the set represented by the additional constraints projected to the remaining (not eliminated) variables. This representation consists of a list of linear systems whose disjunction characterizes the resulting set. In all our computations it turned out that all the linear systems in the disjunction were the same, and so the sets $\text{Force}_{\text{chaos}} - \text{Force}_{\text{constr}}$ are in fact convex in these cases.

The computations of Equi sets require no elaboration.

Remark 1. There is the practical problem that the negation of a $\leq$ -inequality is a $>$ -inequality while standard algorithms only accept closed convex polytopes. To cope with this problem, we approximated $a > b$ by $a \geq b +$ (very small number). We also used a rational approximation to $\sqrt{2}$ in the quant constraints. In both cases, we have repeated the computations with decreasing values of “very small number” until it was obvious where the results converged.
