# Supplementary material for: All-Possible-Couplings Approach to Measuring Probabilistic Context
Source: PLoS One. 2013 May 6;8(5):e61712. doi: 10.1371/journal.pone.0061712 (PMC3646012; doi:10.1371/journal.pone.0061712)
Supplement: Text S5 — Computations for constraint. (PDF) [file pone.0061712.s005.pdf]

S5 Computations for $\mathrm{Fit}_{\mathrm{fix}(p)}$ constraint

That $\max \mathsf{S}_0 p$ and $\max \mathsf{S}_1 p$ are contained in and completely fill the triangle $\{(0,0),(1/2,1),(1,1/2)\}$ can be verified by splitting (38) into 64 component cases according as which of the values of $\mathsf{S}_0 p$ and $\mathsf{S}_1 p$ are the maxima, finding the vertices of each component system, and drawing the union of these components in $\max \mathsf{S}_0 p$ and $\max \mathsf{S}_1 p$ coordinates. The triangle is described by

$$\begin{aligned} &2\max \mathsf{S}_0 p - \max \mathsf{S}_1 p \geq 0,\\ &2\max \mathsf{S}_1 p - \max \mathsf{S}_0 p \geq 0,\\ &\max \mathsf{S}_0 p + \max \mathsf{S}_1 p \leq 3/2. \end{aligned} \tag{S5.1}$$

Adding these inequalities to the representation of (38) as linear inequalities according to the definitions of $\max \mathsf{S}_0 \varepsilon$ and $\max \mathsf{S}_1 \varepsilon$, we obtain a 6D polytope $P^{(6)}$ in $(\varepsilon, \max \mathsf{S}_0 p, \max \mathsf{S}_1 p)$-coordinates. In the V-representation of $P^{(6)}$, all vertices have values of $\max \mathsf{S}_0 p$ and $\max \mathsf{S}_1 p$ in the set

$$\{(0,0),(1/4,1/2),(1/2,1/4),(1/2,1),(1,1/2)\}. \tag{S5.2}$$

It follows that every edge of the polytope projects to one of these 5 points or to a line connecting two of them. Consequently, as $(\max \mathsf{S}_0 \varepsilon, \max \mathsf{S}_1 \varepsilon)$ changes within any triangle T formed by these lines, the cross-section $P^{(4)}_{(\max \mathsf{S}_0 \varepsilon, \max \mathsf{S}_1 \varepsilon)}$ of $P^{(6)}$ retains its structure (face lattice) while its coordinates change as affine functions of $(\max \mathsf{S}_0 \varepsilon, \max \mathsf{S}_1 \varepsilon) \in T$. It follows that the volume of $P^{(4)}_{(\max \mathsf{S}_0 \varepsilon, \max \mathsf{S}_1 \varepsilon)}$ is a polynomial of $(\max \mathsf{S}_0 \varepsilon, \max \mathsf{S}_1 \varepsilon) \in T$ of at most degree four. The coefficients of these polynomials were obtained by fitting unconstrained degree 4 polynomials to the exact volumes $\mathrm{Vol}^4\left(\mathrm{Fit}_{\mathrm{fix}(p)}\right)$ for $(\max \mathsf{S}_0 p, \max \mathsf{S}_1 p) \in \{0, .01, .02, \ldots, 1\}^2$. It turns out that the coefficients change only if either of the differences $\max \mathsf{S}_0 p - 1/2$ and $\max \mathsf{S}_1 p - 1/2$ changes its sign. In all cases the fit is perfect for the number of points far exceeding the number of coefficients, confirming that the computations are correct.
